# How to Construct, Conduct and Analyze an Exercise Training Study?

**DOI:** 10.3389/fphys.2018.01007

**Published:** 2018-07-26

**Authors:** Anne Hecksteden, Oliver Faude, Tim Meyer, Lars Donath

**Affiliations:** ^1^Institute of Sports and Preventive Medicine, Saarland University, Saarbrücken, Germany; ^2^Department of Sport, Exercise and Health, University of Basel, Basel, Switzerland; ^3^Department of Intervention Research in Exercise Training, German Sport University Cologne, Cologne, Germany

**Keywords:** RCT, longitudinal, exercise trial, intervention, statistics, study design, analyzing, reporting

## Abstract

Randomized controlled trials (RCTs) can be regarded as gold standard in investigating dose-response and causal relationships in exercise science. Recommendations for exercise training routines and efficacy analyses of certain training regimen require valid data derived from robust RCTs. Moreover, meta-analyses rely on RCTs and both RCTs and meta-analyses are considered the highest level of scientific evidence. Beyond general study design a variety of methodological aspects and notable pitfalls has to be considered. Therefore, exercise training studies should be carefully constructed focusing on the consistency of the whole design “package” from an explicit hypothesis or research question over study design and methodology to data analysis and interpretation. The present scoping review covers all main aspects of planning, conducting, and analyzing exercise based RCTs. We aim to focus on relevant aspects regarding study design, statistical power, training planning and documentation as well as traditional and recent statistical approaches. We intend to provide a comprehensive hands-on paper for conceptualizing future exercise training studies and hope to stimulate and encourage researchers to conduct sound and valid RCTs in the field of exercise training.

## Introduction

The principal study types available for investigating the effects of exercise training range from retrospective epidemiological and cross-sectional research to prospective controlled exercise training trials. The latter can be considered a gold standard to elucidate causal and dose-response relationships in sport specific research, providing the highest level of evidence. Consequently, longitudinal designs with at least two groups, two repeated measures and a randomized allocation of participants are basically required for many research questions. This corresponds to a randomized controlled (training) trial (RCT). Beyond general design, researchers are faced with various conceptual challenges and pitfalls including characteristics of the interventional approach and control condition (e.g., inactive control, work matched control conditions, treatment as usual, social gathering etc.), reliable and valid outcome measures, study population and sample size as well as the statistical approach for data analysis. Importantly, these aspects are tightly interconnected and have to fit with one another. For instance, the conception of the study design determines statistics and study power. Therefore, the whole study “package” has to be thoroughly constructed and outlined prior to the start.

This manuscript addresses critical aspects of the design, realization and analysis of exercise training studies. A variety of conceptual aspects and their interrelations will be discussed in the light of traditional and recent methodological considerations. We are mainly focusing on factors specific to exercise training studies as compared to other e.g., pharmaceutical interventions (for instance, challenges of blinding, standardization and wash-out, low “*n*” in elite athlete studies, etc.). The manuscript intends to provide a concise, structured and comprehensive presentation of sport specific aspects and their interrelations rather than on comprehensively covering the entire theoretical background and can be regarded as a hands-on paper for developing, understanding and applying specific study designs. We deliberately do not deliver solutions to specific issues regarding a particular study “package.” Instead, we aim to sensitize the interested reader to issues and opportunities which are particularly related to exercise science research. For gaining more detailed insight into specific aspects, we would like to refer to the cited literature.

## Study Design

### General Design

#### Randomized Controlled Trials

Randomized controlled trials (RCTs) are considered the gold standard for evaluating interventions in biomedical research. Well designed and conducted RCTs provide highest evidence level on the efficacy of healthcare interventions (Moher et al., 2010; Thiese, 2014). Trials with inappropriate methods are associated with a high risk of bias (Moher et al., 2010). Thereby, proper and transparent reporting of all relevant methodological issues of an RCT is crucial (Altman et al., 2012). With regard to adequate reporting of RCTs the CONsolidated Standards Of Reporting Trials (CONSORT) Statement has been developed (Schulz et al., 2010). CONSORT is a 25-item checklist to standardize the reporting of key elements of randomized trials (Vohra et al., 2015). Exercise training interventions are complex interventions which are not appropriately and completely addressed by the CONSORT checklist. Therefore, in extension of CONSORT, the Consensus on Exercise Reporting Template (CERT) has been recently developed and may provide a valuable supplement to report and document randomized exercise trials (Slade et al., 2016).

As reflected in the acronym, RCTs are characterized by two key elements: a control group and randomized allocation of participants to two or more study arms. Additional elements such as predetermined outcome measures and blinding are considered crucial for the quality of an RCT and the internal validity of inferences.

The control group provides a proxy for what would have happened to the participants in an experimental group if they had not received the intervention. Pre–post changes in the experimental group may then be directly compared to changes in the control (comparator) arm to gauge the effects of the intervention (Senn, 2009). By contrast, with an uncontrolled design observed changes are generally attributed entirely to the intervention (assuming that nothing would have changed without it). Different types of time- and learning-effects are alternative explanations for observed changes which may be unmasked by comparison to a control group (Hecksteden et al., 2013). Obviously, the value of the control group in leaving the intervention as the only plausible explanation critically depends on its similarity to the experimental group. This concerns baseline characteristics of participants that should not relevantly differ in both group as well as adequate flow through the study (except for the intervention). Other design features such as randomization and blinding aim at ensuring this similarity between groups.

The proper assignment of participants to the study arms is an important aspect of the trial design. Basically, group allocation should be based on chance, thereby, minimizing the risk of selection bias due to differences in group characteristics (e.g., in the primary endpoint and/or anthropometric or demographic data) (Senn, 1995, 2013; Moher et al., 2010). Two main aspects are particularly important in this regard to prevent correct anticipation of future assignments by anybody involved in the trial: (i) an unpredictable allocation sequence must be generated and (ii) this sequence must be concealed until assignment (Moher et al., 2010). It is important to note that randomization should be done by an independent investigator, who is not directly involved in the testing and intervention. Ideally, the researcher, who is running the randomization procedure, works with the fewest information necessary and is only delivered with coded data.

Simple or pure randomization (i.e., group allocation with a 1:1 ratio based on coin toss) works well in large samples as there is a high probability that potential confounders (i.e., age, gender or current performance or physical activity level) are evenly distributed in all study arms. Random allocation can also be done in several more sophisticated ways in order to ensure a balanced distribution of participants’ characteristics which are known to be potential confounders regarding the main study outcomes (Moher et al., 2010). The most prevalent amendment to simple randomization is stratification.

Stratification can be used to ensure that groups are balanced with regard to particular characteristics of participants (strata), which likely affect intervention outcomes. When appropriate stratification according to pre-defined strata (in training studies, for instance, age, gender, baseline physical activity level or study center in case of multi-center studies) is conducted, the number of participants for each study arm is closely balanced within each stratum. A further method for group allocation, which is not actually a random approach, is minimization (Pocock and Simon, 1975; Treasure and MacRae, 1998; Altman and Bland, 2005; Moher et al., 2010). Applying this approach, the first participant is truly randomized. Each subsequent participant is allocated to a treatment or control group in order to minimize the imbalance on selected pre-defined factors, which are assumed to be potential confounders. The number of prognostic factors which can be incorporated is larger in minimization as compared to stratified allocation (Scott et al., 2002). Minimization is advantageous when small groups should be closely matched with regard to relevant participant characteristics. Minimization may not eliminate bias on all known and, particularly, unknown confounders, but is an acceptable alternative to randomization and by some authors considered superior (Treasure and MacRae, 1998; Scott et al., 2002; Altman and Bland, 2005; Moher et al., 2010).

Another important aspect is the adaptivity of randomization to drop outs. In other words: Does the slot of subjects dropping out of the study re-enter the randomization procedure? If the proportion of drop outs differs between study arms, this offers obvious benefits regarding balance in the final sample. An advantage which is particularly relevant if subject number is limited. Moreover, while such a feedback loop seems counterintuitive bearing the idea of “sealed envelopes” in mind, adaptive randomization is deemed admissible if properly conducted by an external scientist (Food and Drug Administration, 2010; Rosenberger et al., 2012).

#### Alternative Study Designs

Conducting a robust RCT is not always possible and in some instances not even the appropriate approach. Particularly in exercise science, studies are frequently conducted in club, school, hospital, or community settings, respectively, and large trials may require a multicenter approach. In all these cases, participants are packed into clusters, in which observations are not necessarily independent and tend to be correlated. In order to avoid contamination, i.e., the unintentional transfer of intervention (elements) to other members of the cluster which are actually assigned to the control group, the clusters should serve as the units of randomization (Campbell et al., 2012; van Breukelen and Candel, 2012). Cluster-randomization affects the power and, consequently, increases the necessary sample size of a trial and clustering has to be considered as a covariate when analyzing the data (van Breukelen and Candel, 2012).

In some instances, it might be necessary to evaluate areas of uncertainty prior to conduct a definitive RCT. In such cases, a pilot randomized trial is the means of choice (Eldridge et al., 2016). Meanwhile, a variety of competitive funding bodies are claiming for those pilot data. The primary aim of a pilot study is commonly feasibility, which affects the methodology used in a pilot trial. Assessments and measurement procedures should be chosen according to the aims of the pilot trial, not necessarily the definitive RCT. The sample size in pilot trials is usually lower compared to the final RCT, but should also be rationalized. Hypothesis testing regarding intervention efficacy is generally not indicated as the pilot trial is likely underpowered for this purpose. However, the standards for conducting and reporting of definitive RCTs also apply to pilot studies (Eldridge et al., 2016).

Beyond the typical parallel group RCT, a randomized crossover trial is another option to implement a control condition and random allocation of participants. Therefore, randomized crossover studies may be categorized in the RCT design family (Hopewell et al., 2010). In contrast to the parallel group RCT, each study participant performs both study conditions in a randomized order, i.e., each person serves as her or his own control (Thiese, 2014). A crossover RCT begins similar to a traditional RCT, but after the first intervention period the participants cross over to the other study arm. Between both periods usually a wash-out period is interposed in order to ensure that baseline data are comparable. Particularly in training studies, this can be a challenge as training effects usually need considerable time to diminish or the intervention under investigation has to be incorporated in practical routines and/or periodized training schedules (Faude et al., 2013, 2014). The reversibility of a treatment effect is a necessary prerequisite for applying crossover designs and determines the length of the wash-out period. This is a particular challenge if valid data on detraining effects are not available. Potential carry-over effects have to be considered during data analysis. When using a crossover design, no participant is excluded from the promising treatment under investigation and sufficient statistical power can be achieved with fewer participants. The effort needed by each participant, however, is greater.

Robust RCTs are able to reliably predict intervention efficacy on a group level. Prediction of benefits and harms of a particular intervention on the individual level, however, is not possible without a high amount of uncertainty. In such cases, *N*-of-1 trials might be the appropriate means of choice (Hecksteden et al., 2015; Senn, 2015; Vohra et al., 2015). When the recruitment of a large enough population is limited, *N*-of-1 trials might be the ideal methodological alternative. In exercise science and sports medicine, such scenarios are frequent in high performance sports, with the available elite population being naturally limited, in patients with rare diseases or when strong inclusion and exclusion criteria must be applied to arrive at homogeneous samples, limiting the number of eligible participants. A prerequisite for *N*-of-1 trials is a relatively quick onset of action and termination after discontinuation of the intervention which can make such scenarios difficult with regard to specific training adaptations. *N*-of-1 trials can be conducted as single or multiple crossovers, comparing a treatment against no or another treatment within one individual serving as her or his own control (Vohra et al., 2015). Thus, potential confounding is eliminated given an appropriate wash-out period is applied in order to ensure similar baseline conditions for the treatment arms. A typical design is treatment – withdrawal – treatment – withdrawal (ABAB design). Multiple crossovers increase the confidence of the obtained results.

In some instances, uncontrolled or non-randomized study designs may be justified. Uncontrolled trials are less expensive, more convenient and faster to conduct than RCTs (White and Ernst, 2001). When applying a single arm pre–post design without a control arm, however, there is a considerable risk that other factors than the intervention (e.g., familiarization, changes in lifestyle or activity behavior in addition to the exercise intervention, social desirability, regression to the mean) may also account for at least a part of the observed changes. Uncontrolled studies may be justified for pilot studies in order to get insight into associations between variables or expectable effect sizes of the intended intervention or regarding the feasibility of an intervention or specific treatment components (White and Ernst, 2001). Uncontrolled trials, however, should be always interpreted very carefully as it is impossible to exclude that any changes, which occurred during the intervention period, would not have occurred without the intervention. Similarly, non-randomized trials include a control group or condition, but the allocation to the group is not due to chance but likely to the preferences of the participant which may affect the efficacy of the program and, thus, leading to a biased interpretation of the intervention. For instance, in injury prevention research, allocation to an intervention group performing an injury prevention program or to a control group doing their normal training routine based on the willingness of the team coaches, is likely biased as coaches who are willing to do the program are more aware of the injury problem and, hence, may also have implemented other measures to reduce injuries. The efficacy of the program may, therefore, be overestimated in non-randomized designs (Mandelbaum et al., 2005; Gilchrist et al., 2008). In summary, whenever possible an appropriate comparison group should be included in any interventional study and group allocation should be done randomly.

Whereas RCTs are considered the gold-standard for establishing cause-effect relationships and clinical decision making, there are also some disadvantages regarding the transferability of the results to sports and clinical practice (Wilkerson and Denegar, 2014). For instance, in RCTs frequently strong inclusion and exclusion criteria are used to increase statistical power and the precision of the intervention effect estimate. Furthermore, RCTs provide an estimate of the efficacy of an intervention under ideal, strictly controlled conditions, particularly with regard to the administration of the intervention. The effectiveness, i.e., when administering the intervention under real, more natural circumstances, of a particular treatment cannot be reliably evaluated by an RCT.

Prospective cohort studies may provide a feasible, well justified and useful alternative or complement to traditional RCTs and can result in improved decision making when guiding individual exercise training scenarios (Thiese, 2014; Wilkerson and Denegar, 2014). A large cohort is initially recruited and desired baseline parameters, e.g., physical activity or fitness and/or health-related physiological and laboratory markers, are assessed. The cohort will be followed for a pre-defined period of time and individual exposure to a specific training mode will be documented in order to analyze changes in the outcomes of interest in the group which was exposed to training compared to the group which was not exposed. It is possible to analyze a large amount of possible moderators, mediators and confounders allowing for heavily multivariate analyses, given that the sample is large enough. In addition, generalizability of the results can be better as studies usually are conducted in more naturalistic settings.

A summary of design types in exercise training research is presented in **Table 1**.

**Table 1:** Summary on relevant design types in exercise training research.

Design type	Comments
Randomized controlled trial (RCT)	• The gold standard for evaluating the efficacy of exercise training interventions
	• Characterized by two key elements: a control group and randomized allocation of participants to two or more study arms
Cluster-RCT	• Appropriate design type when participants are packed into clusters (e.g., study centers, schools, clubs, and hospitals) to avoid contamination within clusters
Pilot-RCT	• Means of choice to evaluate feasibility [e.g., regarding intervention (elements), training application, procedures] in order to prepare for a definitive RCT
Randomized crossover trial	• Each study participant performs both the intervention and control condition and, thus, serves as her/his own control, thereby, saving “*n*”
	• An appropriate “wash-out” period is a major challenge in exercise training studies
*N*-of-1 trial	• Enables to evaluate the benefits and harms of an intervention of interest on the individual level
	• Particularly relevant, when the population of interest is small (e.g., elite athletes, patients with rare diseases)
Uncontrolled/non-randomized trial	• Justified as pilot or exploratory trial to get preliminary data regarding relevant design decisions of a definitive RCT
	• Should be interpreted very carefully
Prospective cohort study	• Enables an investigation of exercise routines under more naturalistic circumstances in “real” life
	• Enables the analysis of a large amount of possible moderators, mediators, and confounders

### Critical Specifications

#### Study Aim and Hypothesis

When a general design type has been selected for a particular study question, critical design specifications have to be defined. Initially, a specific study aim together with the study hypothesis must be clearly formulated prior to the start of the trial. In this regard, it is very important to distinctly state whether it is hypothesized that a new intervention (treatment under evaluation) is superior to a reference treatment or control condition (superiority hypothesis) or that a new intervention is similarly efficacious or not worse compared to the reference or current gold standard treatment (equivalence or non-inferiority hypothesis). Whereas studies usually aim to evaluate the superiority of one treatment over another one, equivalence or non-inferiority trials are also justified under particular circumstances (Piaggio et al., 2012). This is, for instance, the case if the new intervention has some advantage other than increased efficacy, such as better availability, better cost-effectiveness, less invasiveness, fewer adverse effects (harms) or easier administration. Equivalence or non-inferiority trials differ from superiority trials with regard to methodological and statistical considerations, for instance, by *a priori* defining an equivalence region (Piaggio et al., 2012; Lakens, 2017; Dixon et al., 2018; Lakens et al., 2018). For a deeper insight into this issue we refer to the cited literature.

#### Study Outcomes

Once the specific study question(s) and hypotheses have been formulated, primary and secondary study outcomes have to be defined. The chosen outcomes should be closely related to the study aims and hypothesis with a minimal amount of primary outcome(s) to answer the main study question. Secondary and tertiary outcomes should be defined in order to support the main findings regarding training efficacy or to give insights into potential mechanisms of training adaptations. Particular emphasis has to be put on this aspect when participants are to be classified as responders and non-responders according to observed changes in one or more outcome measures because an individual’s “response” may be outcome specific (Scharhag-Rosenberger et al., 2012). Moreover, if several parameters are to be jointly considered in the response classification, decision rules have to be explicitly fixed during the planning stage of the trial (Hecksteden et al., 2015).

Outcomes should be chosen according to clarify theoretical or evidence-based rationales. Strong outcomes in exercise and health sciences are, for instance, mortality, morbidity or injuries as these are “hard” endpoints with obvious clinical and practical relevance. However, to analyze such outcomes, usually large samples are needed. “Hard” endpoints such as mortality or hospital admission are commonly used in epidemiological research. Conducting an RCT with such outcomes is a challenging endeavor (Shiroma and Lee, 2018). Frequently, surrogate parameters such as maximal oxygen uptake (VO_2_max; as a surrogate of cardiovascular fitness or health) or maximal voluntary contraction strength (as an indicator of muscular fitness or health) are used in RCTs due to their better feasibility and relevance for sports and clinical practice. In such instances, it is important to rationalize the clinical or practical relevance of the chosen parameter. For example, in a homogeneous sample of high-level athletes VO_2_max is a poor indicator of endurance capacity (Meyer et al., 2005). Lactate threshold and running economy might be more suitable under specific circumstances (Coyle, 1995; Faude et al., 2009), but simulated sport-specific time trial performance is probably the best choice (Abbiss and Laursen, 2008; Currell and Jeukendrup, 2008). Particular value when selecting appropriate study outcomes should be placed on the reliability and validity of the chosen measures (Atkinson and Nevill, 1998; Hopkins, 2000; Currell and Jeukendrup, 2008). Knowledge of the inter- and intra-individual variability, i.e., the reliability of a particular assessment tool, is necessary to correctly interpret and detect intervention effects based on changes in performance or physiological parameters. Being aware of the test reliability enables a researcher to determine the boundary between true changes and differences which may result from random variability only, i.e., to determine the “minimal detectable change (MDC)” within a specific test (Atkinson and Nevill, 1998; Hopkins, 2000; Haley and Fragala-Pinkham, 2006; Hecksteden et al., 2015).

#### Study Population and Sample Size

A further prerequisite for a robust exercise training study is the appropriate choice of the population under investigation. Many exercise training studies are conducted using physical education students as participants, simply as this is the population which is directly and most easily available for sport science researchers. Whereas this choice might be justified in some instances, in most cases it is not. For instance, resistance training adaptations in sports students are unlikely the same as in high level resistance athletes with many years of specific training experience and an already extraordinary performance level which does not leave much room for further improvements (ceiling effect). Moreover, interventions which are effective in a healthy, young and active population do not necessarily apply to old and frail people or people with specific diseases. Consequently, the population under investigations should be closely matched to the study aims and hypotheses and the researchers should *a priori* determine the eligible population and define corresponding inclusion and exclusion criteria. Thereby, all inclusion criteria have to be fulfilled by a participant to be eligible. If at least one exclusion (or non-inclusion) criterion is fulfilled the person must be excluded from study participation.

**FIGURE 1:**
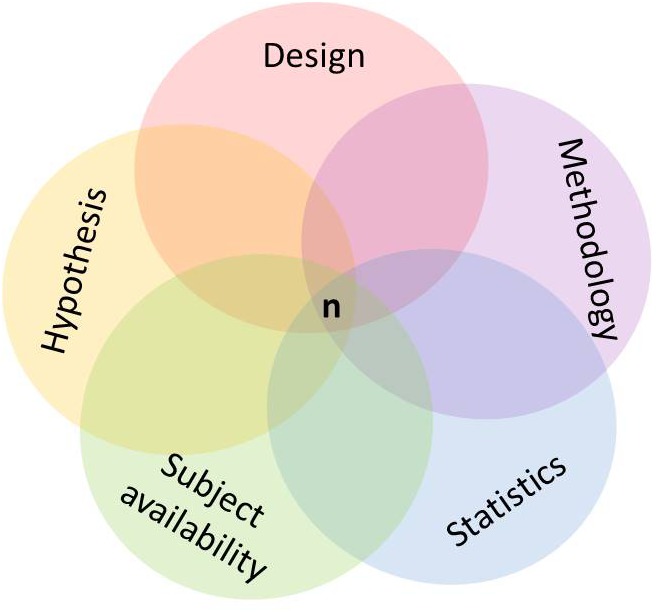
Conceptual aspects and their interrelations in the design of exercise training studies.

An important issue regarding the participating population refers to the appropriate sample size. From an ethical perspective, it is important to study a sample which is large enough to detect an effect with an acceptable accuracy (Hopkins, 2006). Is the sample size too large, people are unnecessarily exposed to and waste resources with an intervention which potentially can be risky, harmful, or painful. In this case, small, but clinically or practically irrelevant effects might be detected as significant. Is the sample size too low, resources will be wasted with a high risk of failing to detect a relevant effect. Sample size estimation is usually required before the study protocol is submitted to a funding institution or an ethics committee. In order to estimate an appropriate sample size, several issues have to be considered a priori, i.e., during the process of study planning. Justification and reporting of the required sample size should be done carefully and honestly. Besides the study design, several parameters have to be considered, for example, the number of main outcomes, the smallest clinically or practically worthwhile effect, types I and II error rates, the baseline variability of the main outcome parameter as well as the statistical approach for data analysis (Batterham and Atkinson, 2005; Hopkins, 2006; van Breukelen and Candel, 2012). Consulting with a statistician is recommended at this stage, particularly when projecting a complex study design. Failure to correctly consider one or more aspects may irremediably preclude study success while, on the other hand, fine tuning the methodology and design “package” reduces the burden imposed on researchers and participants and increases the likelihood of meaningful results (**Figure 1**).

#### Choice of Control “Treatment”

When it comes to the decision on the appropriate control arm, there are several options, which have to be considered. The final decision depends on the complete design “package” of the particular study. From the perspective of internal validity, a control group receiving the recommendation to maintain their usual (e.g., inactive) habits might be considered the best choice as the main purpose of the control group is to control for what might have happened to the intervention participants if they had not received the treatment. Such a choice, however, can have several limitations. Completely inactive participants are not necessarily reflecting the “real world” setting. Most people are aware – or become aware within the study setting – of the beneficial effects of physical activity or exercise training and, therefore, complete inactivity does not reflect externally valid conditions and can be regarded ethically doubtful in some populations (e.g., patients or seniors) when a potentially beneficial intervention is denied. It might be considered to inform or educate the control participants on the beneficial health effects of physical activity and encourage them to follow general physical activity recommendations (Chodzko-Zajko et al., 2009; Garber et al., 2011). Another issue with inactive controls is the missing attention, social contact or study-directed activities which may raise expectations and could have a beneficial effect by itself (Lindquist et al., 2007). This should be considered, particularly, in elderly populations where social separation is a relevant problem and being active together with other seniors or the researchers might improve the individual abilities and quality of life by itself. For instance, arranging regular meetings where the control group members meet to play cards and chat, allowing for social contacts, while being physically inactive, might be an option (Donath et al., 2014). Furthermore, participants might refrain from study participation or they drop out during the study period, because they were assigned to the control group and do not receive the anticipated intervention. Such dissatisfaction with group assignment can also lead to unintended and uncontrolled lifestyle changes in control group members potentially leading to considerable confounding and a decrease in power (Hertogh et al., 2010). To overcome such scenarios there are mainly two options. First, a wait-list control group, which receives the intervention after having served as an inactive control, might be applied. This option has the advantage that there is potentially no change in the lifestyle habits of the control group members as they are provided with the intervention at a later time. However, with long intervention periods this approach becomes challenging and expensive and might be regarded unethical (e.g., in patients or seniors). Second, an active control group, which engages in activities related to the research setting and, thus, accounting for potential treatment effects, can be an appropriate choice. Such an active control group can receive health education, social visits or an alternative (but likely ineffective) exercise regimen (Lindquist et al., 2007).

#### Placebo and Blinding

A double blind placebo controlled parallel group design is considered the “gold standard” in biomedical research (Beedie et al., 2015). The placebo is a negative control as it is pharmacologically inert. It is expected that participants in the placebo control arm show a change or response to the investigation, including the response to a therapeutic ritual, to observation and assessment and to the patient-researcher interaction (Sedgwick and Hoope, 2014). Ideally, placebos should be indistinguishable from the actual intervention and, therefore, participants should be unaware whether they receive the placebo or the treatment, allowing the researchers to estimate the “real” treatment effect (Beedie et al., 2015).

In exercise training studies, it is obviously difficult to blind the participant to the intervention as they usually are aware whether they are training or not. In exercise science and sports medicine, placebo controls can be easily applied when the efficacy of ergogenic aids is assessed (Beedie and Foad, 2009; Berdi et al., 2011). Berdi et al. (2011) found that an average overall effect size indicating a placebo effect of 0.4 (95% confidence interval 0.24–0.56) was present in placebo controlled studies analyzing the efficacy of different ergogenic substances. An interesting study was conducted by Broatch et al. (2014) showing that a placebo thermoneutral water immersion resulted in better recovery after high-intensity training compared to pure thermoneutral water immersion. Recovery efficacy was similar for the placebo condition compared to cold water immersion. The placebo effect was facilitated by adding a “recovery lotion” (customary skin cleanser) to the water.

Incorporating an adequate placebo condition is a serious challenge in training studies, is frequently not feasible and it might even be not appropriate (Beedie and Foad, 2009). For instance, applying the placebo effect might be ethically problematic as it may exploit the bond of trust between practitioner and client or scientist and study participant. Furthermore, a nocebo effect may occur, i.e., the (inert) “placebo” treatment induces harmful side effects. Finally, according to the newest version of the Declaration of Helsinki (World Medical Association, 2013), placebo is only acceptable (i) when no proven intervention exists, (ii) based on sound methodological reasons the use of placebo is necessary to determine the efficacy or safety of an intervention or (iii) when the participants are not exposed to additional risks of serious or irreversible harm, because they do not receive the currently best proven intervention. Therefore, an active control arm is usually preferable and the use of placebo has to be seriously justified (Beedie and Foad, 2009; Sedgwick and Hoope, 2014). An active control arm, for instance, can be the current best-practice or best-evidence approach. Alternatively, the clinical standard treatment (irrespective of being best-evidence) or treatment as usual can be used as control arm.

Ideally, randomized trials are conducted with appropriate blinding or masking, i.e., withholding information about the intervention which may affect the study outcomes from people involved in the trial (Moher et al., 2010). Blinding is an adequate means against several forms of bias. Risk of bias is highest in parameters which can be affected by subjective expectations. Blinding can be introduced on different levels of the trial design and is sometimes referred to single-, double- or triple-blinding, although this denotation is subject to variability, misinterpretation, and confusion (Schulz and Grimes, 2002; Moher et al., 2010). Researchers should honestly report the blinding of all people who may be affected by the knowledge of the intervention assignment. These people can be, the participants of the trial, the providers of the intervention (e.g., physicians, therapists, coaches, teachers, etc.), data collectors (i.e., the testing staff) and those who assess the data (Schulz and Grimes, 2002). Moreover, it has been debated that also those people who manage and analyze the data as well as manuscript writers can be blinded, but this is a matter of debate (Moher et al., 2010). Obviously, masking the assignment of the participants to an exercise intervention group or an inactive control group is nearly impossible. In case of parallel group designs with treatment as usual as a control, it is advisable to withheld information on the hypothesized intervention efficacy from the study participants. Similarly, it is often difficult to blind the intervention providers as they have some expertise in the field of research and corresponding expectations regarding intervention efficacy. Exercise training cannot be masked in a way as, for instance, pharmacological or ergogenic substances.

An important level of blinding in exercise science research remains with the data collectors. Testing staff can affect study outcomes in different ways by their (unconscious) behavior and expectations, when knowing the group allocation of an individual. For instance, encouraging study participants during VO_2_max tests or maximal strength assessment can be realized differently depending on whether the participant was in the intervention or the control group. Therefore, blinding of the testing staff to group allocation and uniform and standardized procedures are advisable in order to minimize the risk of bias on the data assessment level.

#### Transferability and Implementation Considerations

If the efficacy of a particular exercise intervention has been established in an RCT, the question arises, whether the intervention can be easily transferred to the real-world setting. In most instances, program efficacy is strongly related to compliance and compliance is likely better in a standardized, controlled setting like in RCTs. When constructing an RCT, researchers should at least consider relevant questions on the transfer and uptake of a particular intervention in practical settings and on how the treatment can be maintained in the long-term after efficacy has been established. Researchers should be aware of and describe possible arrangements to give people access to an intervention after a study has identified a treatment as being beneficial for a particular population. In this regard, it is sometimes recommended to involve participants in the design of research and potentially also in the dissemination of an intervention (Harriss and Atkinson, 2015).

A central framework regarding health program implementation is the RE-AIM (Reach, Efficacy, Adoption, Implementation, and Maintenance) framework and its adaptation to the sports setting, the RE-AIM Sports Setting Matrix (RE-AIM SSM) (Finch and Donaldson, 2010). This concept is specific to the implementation context in community sports and may guide the promotion of prevention programs. O’Brien and Finch (2014) systematically reviewed the scientific literature on the reporting of specific implementation components in team ball sport injury prevention programs using the RE-AIM framework. The authors concluded that there are major gaps in adoption and maintenance in injury prevention research and, consequently, that reporting of the implementation context was insufficient. Thus, it is recommended to consider the implementation context already when designing an exercise intervention trial.

#### Ethical and Legal Obligations

Finally, ethical issues and legal obligations have to be considered. Like all biomedical research, exercise training trials must be in line with the Declaration of Helsinki in its latest version from 2013 (World Medical Association, 2013) as well as with current data protection and data security regulations (Harriss and Atkinson, 2015). Importantly, compliance with ethical and legal constraints cannot be decided by the researchers themselves, instead the final protocol has to be reviewed and approved by an appropriate ethics committee (Harriss and Atkinson, 2015). It is highly recommended to publish the study protocol and several scientific journals require an a priori registration in an appropriate trial registry (for a summary of primary trial registries^1^) (World Medical Association, 2013). Harms and serious adverse events must be appropriately and honestly reported. This is a particularly underrepresented issue in exercise science research as studies commonly target the undoubtedly beneficial effects of physical activity and exercise training for fitness and health, but frequently ignore potential side effects and harms of being active (e.g., injuries or cardiovascular events) (Verhagen et al., 2015).

## Exercise Training Interventions

An important issue of the study “package” in exercise training studies is the design of the exercise intervention. In exercise science there are specific considerations related to the applied intervention, which usually differ considerably from interventions in other biomedical areas and are closely linked to the choice of the other “package” components. Particular considerations regarding the design and documentation of exercise interventions are presented in this chapter.

### Comparability of Training Study Arms

When comparing two training modes in terms of efficacy, training characteristics such as setting, mode and load should be thoroughly considered and documented (**Table 2**). For example, if the effects of interval training on improving maximal oxygen uptake are compared to continuous aerobic endurance exercise equicaloric exercise loads are required (Helgerud et al., 2007). Otherwise, it is hardly possible to elucidate whether the interval pattern or the difference in energy expenditure accounts for potential differences in VO_2_max adaptations. Estimating the caloric expenditure in highly intense exercise bouts, however, is a tremendous challenge, as excess carbon dioxide during intense exercise notably affects the calculation of the caloric expenditure. An alternative perspective might be to induce comparable effects with lower volumes. For instance, this has been shown with sprint interval training and its effects on cardiovascular function (Gibala et al., 2006; Burgomaster et al., 2008).

**Table 2:** Relevant training characteristics that should be taken into account prior to the start of a training intervention in order to determine the external load of exercise training studies.

**Training setting**	Home based	Institutional	Individual	Group based	Inpatient	
**Training mode**	Endurance	Strength	Balance	Speed	Flexibility	Mixed
**Training load**	Volume	Time	Frequency	Repetitions	Intensity	Response

Neuromuscular training is much easier to match properly as the cardiorespiratory or caloric demands are comparatively low. Repetitions, times and loads can be feasibly objectified. The total volume and frequency should be monitored and reported. For example, comparing whether explosive strength training (high movement velocity at lower loads) is favorable than traditional strength training (moderate movement velocity at higher loads) in terms of reducing fall risk factors requires similar training loads. Thus, the load should be precisely calculated for each training session. Thereby, mood states, perceived exertion, heart rate response are complementary means to monitor internal response compared the externally equivalent load. If multimodal training approaches such as agility training that integratively triggers cardiovascular and neuromuscular pathways are considered in preventive exercise training, workload matching is more challenging and should be further investigated compared to separated strength, endurance and balance training (Donath et al., 2016b).

### Exercise Training Characteristics

The duration of exercise training interventions with a neuromuscular or cardio-circulatory focus can vary between a couple of weeks and years (Donath et al., 2016a; Rodrigues et al., 2016; Faude et al., 2017). Studies lasting one year or longer are rare. The majority of available exercise training studies in clinical and non-clinical populations typically range between 4 and 12 weeks, which is frequently justified by feasibility and economic reasons. Consequently, training concepts generally rely on studies with relatively short interventional periods. Longitudinal studies with follow-up periods of several months or years are urgently required. For instance, the lack of transfer effects in balance training studies, which has been recently reported (Giboin et al., 2015; Kummel et al., 2016; Donath et al., 2017), is based on training studies lasting up to 12 weeks. It might be possible that transfer effects will occur later during the long-term training process. Furthermore, it seems that the overall training volume constitutes the main trigger for training adaptations, especially resulting from endurance training (e.g., regarding mitochondrial function) (Granata et al., 2016). Therefore, it is reasonable to assume that higher total training volume lead to larger training effects. A variety of training studies suggest that 40–50 sessions over several months induce robust exercise training effects in neuromuscular domains (Lesinski et al., 2015; Sherrington et al., 2017). Even hard endpoints (e.g., falls, death) benefit more when intervention duration is long and challenge is high. Ultimately, intervention durations should rely on general and individual responsiveness of the respective functional system of interest (e.g., vagal tone, maximal strength, physical activity or fitness, co-contraction) and the annual/seasonal time course of those parameters. For example, if bone mineral content would have an undulating pattern through the year depending on seasonal variations in physical activity patterns and sun exposure, it might be reasonable to spend more interventional efforts during this time and benefit in the more inactive autumn and winter period. Such considerations are mandatorily required prior to the conceptualization of exercise training studies.

Training characteristics such as periodization of training and exercise intensity distribution become likely more important in athletic populations (Seiler, 2010; Stöggl and Sperlich, 2015) and should be particularly considered in training studies in elite populations. Training frequency and intensity are further relevant training characteristics. Few studies investigated differences in training effects depending on frequency and intensity with adjusted total volume in the long term. Interestingly, “weekend warrior” studies indicate that training frequency seems to be a secondary training characteristic in the general population (Meyer et al., 2006; O’Donovan et al., 2017). Exercise training effects do not immediately decrease (reversibility) after exercise cessation depending on age and training state and can maintain for 2–6 weeks after the intervention (Toraman, 2005; Toraman and Ayceman, 2005). Thus, dosage of exercise training need to be justified based on sustainability of effects and potential side effects of exercise training. It is important to consider the potential benefit-risk relation when applying exercise as medicine or preventive means (Verhagen et al., 2015).

**FIGURE 2:**
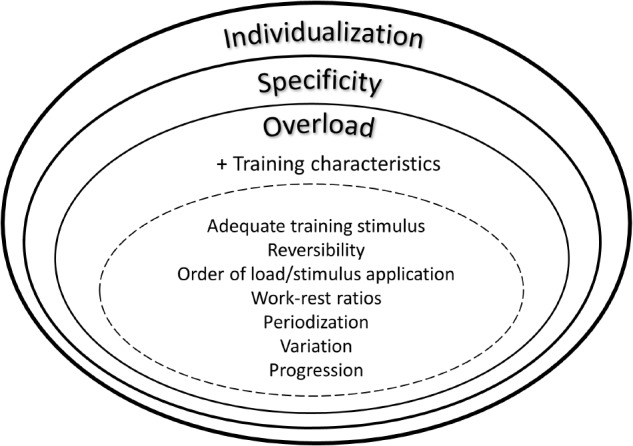
Integrative view on exercise training principles.

Further important issues regarding intervention studies and, particularly, implementation strategies are individual responsiveness, training specificity and overload (**Figure 2**). In this regard a personalized training schedule, based on individual needs, goals, barriers and background can be regarded essential. As a consequence, researchers should also focus on follow up effects and implementation strategies using behavioral change techniques and face-to-face or remote coaching (Foster et al., 2013). Such individualized and tailored exercise training approaches likely lead to sustainable behavioral change. Furthermore, interference effects (e.g., strength training prior to endurance or vice versa) should be carefully considered when conceptualizing exercise intervention studies. In this regard, numerous research has been undertaken during recent years to elucidate interference effects between strength and endurance training on molecular level (Hawley, 2009; Fyfe et al., 2014). These findings do also have impact in the light of general health-related physical activity guidelines that focus on strength, endurance and balance either ways. Thus, the mix of different training stimuli can relevantly affect training adaptations. Another issue in the context of “training variables” is the concept of training progression. To date, only few studies specifically investigated the effects of different progression models on performance or the time course of performance adaptations. Generally, the above mentioned training characteristics (e.g., frequency, intensity, including different work-relief ratios, time, type, volume, **Table 3**) have to be reliably and honestly documented and reported.

**Table 3:** Exercise training characteristics (FITT; Frequency, Intensity, Type, Time).

Training characteristics	Explanation
Frequency	Amount of training sessions per day or week
Intensity	Absolute (velocity, weight, and power output) and relative (percentage of VO_2_max, HR_max_, 1RM) load
Type	Movement execution and position as well as functional domains (e.g., cardio-circulatory or/and neuromuscular)
Time	Duration and repetitions of one or multiple training stimuli and exercises (Volume = Frequency × Time)

*VO_2_max, maximal oxygen uptake; HR_max_, maximal heart rate; 1RM, one repetition maximum.*

### Training Monitoring and Documentation

Monitoring of exercise training includes external (e.g., distance, power, and velocity) and internal (e.g., ratings of perceived exertion, heart rate, blood lactate concentrations) loads. Training volume is mainly linked to external loads and adaptability is also related to internal loads and responsiveness, respectively. Both components should adequately be taken into account and recorded. A variety of subjective and objective instruments to monitor exercise training loads are available. Those systems are feasible to assess individual acute and chronic response to exercise training and the course of recovery (Hopkins, 2015; Bartlett et al., 2017). Since no “one-size-fits-all” gold standard for training load monitoring exists (Lambert and Borresen, 2010), validated (e.g., population, discipline, type of exercise) and reliable methods should be rationalized with regard to the specific study background and appropriately selected. Internal and external training loads can be feasibly documented using web-based and paper-pencil diaries. This is important in order to check training compliance. Furthermore, detailed training documentations potentially enable subgroup analyses in larger cohorts or considerations on responsiveness to the training protocol and constitutes important quality criteria of exercise training trials.

Within recent years, particularly subjective response to training has been emphasized to play an important role in training adaptations and performance enhancement. Also studies on affective valance or enjoyment have been increasingly applied in health-related exercise training research. It seems reasonable to assume that particularly subjective perceived efforts or valance notably affect compliance and adherence to exercise training in the long run. Besides independent assessment of internal and external load, Banister and Calvert (1980) combined exercise duration with a weighted heart rate response. This concept has been proven for continuous endurance exercise and has its limitations within intermittent sports (e.g., soccer and basketball). Promising future concepts can be the monitoring of the response of perceived exertion and the integration of wearables. Those methods should be carefully applied in the intersection between athletes and coaches based on best available evidence in order to predict performance development (Foster et al., 2017).

### Compliance and Adherence

From a methodological and dose-response perspective, compliance and adherence are important aspects of exercise training studies. There is a distinct difference between compliance and adherence. Compliance describes the degree to which an individual conforms to the prescribed dosage of an intervention and is necessary in efficacy trials. Adherence refers to a process which is affected by the environment and social contexts and, therefore, relevant in effectiveness studies (McKay and Verhagen, 2016). Generally, adherence is considered a patients’ or a person’s agreement to the recommended exercise training regimen and treatment, respectively (World Health Organisation, 2003). This is particularly important in non-intrinsically motivated participants. In these individuals, the likelihood to comply with or adhere to the exercise regimen is comparatively low. Participants should be seen as active partners. Thus, knowledge and beliefs on the importance of a respective exercise training for, e.g., disease prevention or performance enhancement approach should be educated and communicated. It is well known that poor adherence is a striking issue in the treatment of chronic conditions. Especially persons with the poorest physical, cognitive and psychological functional abilities representing the part of the population at highest burden of disease do not seem reachable in multifactorial risk-based interventions (Sjosten et al., 2007). Resulting consequences are poor health outcomes and increasing health care costs. Adherence is a powerful modulator of health care system effectiveness and, thus, more balanced efforts should be made to improve adherence instead of only developing specific treatment strategies (Haynes et al., 2008). This is particularly important since a lack of adherence or exercise training cessation may lead to detraining effects (Sherrington et al., 2011).

Only few studies are mainly conceptualized to investigate the interrelation between compliance and adherence and the intervention effect. However, such studies are urgently needed. The majority of available studies merely run sub-analyses on the interrelation between intervention effects and compliance and, as a consequence, does not find associations between compliance and intervention efficacy (Simek et al., 2012; McPhate et al., 2013). As a consequence, RCTs should at least aim at providing “intention-to-treat” and “as treated” comparisons. Intention-to-treat analyses can be merely conducted when participants agree to posting-testing, although dropping out of the intervention. Reasons for drop-outs and a decline from post-testing should be provided. Drop outs can be a systematic response to the intervention regimen and should be necessarily followed up (e.g., too intense, discomfort, inadequate coaching, logistical efforts, etc.). In large cohorts, also the relationship between attendance rates and training adaptations can be computed. Available drop out-rates in interventions studies should also be considered during the sample size estimation. Depending on the population, training mode, volume and intensity, drop outs normally vary between 20 and 50%. Future research should consider compliance and adherence (study design and data analysis), particularly with regard to the implementation of efficacious exercise-based health care interventions. A multidisciplinary approach toward adherence is needed (coordinated action from health professionals, researchers and policy-makers) (World Health Organisation, 2003).

## Data Analysis and Interpretation

Subsequent to the experimental phase, the last steps toward (and hopefully leading to) answering the initial research question consist in analyzing the obtained dataset and interpreting the results. On the bottom line this means trying to make inferences on real-world circumstances based on the data collected. There is considerable controversy regarding appropriate inferential frameworks and statistical techniques and some of the key points in this debate will be addressed later in this paragraph. However, there are also some unequivocal aspects which are dictated by the logic of the trial design. These basic rules will be presented in the following paragraph in (roughly) descending order or indispensability.

The standard case is that (i) the research question concerns the mean efficacy of an intervention (e.g., training induced increase in VO_2_max or maximal strength) and that (ii) a RCT (the current criterion standard for this kind of research question) has been conducted. We shall now harness the specific characteristics of our design to maximize validity of our inferences – that means to zoom in on intervention efficacy by ruling out pertinent alternative explanations.

### Basic Rules for Analyzing an RCT

The analytical techniques mentioned in this paragraph represent the mainstream hypothesis-testing approach to inferential statistics and are applicable for outcome measures which are ratio scaled and normally distributed. Please compare later paragraphs for a brief discussion of alternatives.

The absolutely essential rule for the analysis of an RCT is that assessment of intervention efficacy should be based on direct between-group comparison (Senn, 2009). Comparison to changes in the control group lends support to causally ascribing observed changes in the experimental group to the intervention. Consequently, the question to be answered is whether the change in an outcome (e.g., aerobic capacity, maximal power, and balance ability) in the intervention group differs from the change in the control group. Importantly, the between-group comparison has to be made directly and not indirectly by assessing pre–post changes separately within each group. This basic rule is dictated by the rational of the trial design and applies regardless of the inferential framework and statistical approach. Within the mainstream approach of hypothesis testing, the basic option to test the difference in change-scores between groups is to conduct a *t*-test for independent samples. Alternatively, repeated measures analysis of variance (ANOVA) may be employed with at least the repeat factor (pre- vs. post-test), a group factor (intervention vs. control) and the interaction between the two (which is the main effect of interest).

Moreover, potential baseline imbalances between groups should be considered (Senn, 1995, 2009). In a controlled trial, randomization (following more or less complex rules as explained above) is primarily employed to ensure equal distribution of covariates between groups in order to rule out alternative explanations and support causal inferences. However, perfect baseline balance is unlikely. Therefore, relevant moderators of intervention efficacy should be considered during data analysis. Importantly, whether or not to consider additional influencing factors, and if so, which ones, should be decided in advance (based on their presumed relevance as moderators of intervention efficacy) and not during analysis (based on observed differences at baseline) (Senn, 1995). Generally, following the “rule of the initial value,” at least the baseline value of the outcome measure itself should be included. These recommendations are supported by many strong arguments, which are exposed comprehensively by Senn (1995). For the non-statistician, the most compelling one in favor of doing so may be the surprisingly low “cost” in terms of subject number (Senn, 2013). The consequent type of (standard inferential) analysis is analysis of covariance (ANCOVA). Alternatively, covariates and categorical predictors may be included in a mixed model which otherwise comprises at least subject identity as random effect as well as time and group as fixed effects. The mixed model approach is particularly useful if the outcome measure has been determined on more than two time points.

As mentioned above, one of the initial steps in constructing a training trial consists in formulating the hypothesis as specifically as possible. The control group in exercise training trials does either not receive any intervention or the current best-practice treatment. In most instances, it is expected that the average change over the intervention period is superior in the intervention group as compared to the control group (e.g., larger increase in VO_2_max). Therefore, we generally have a directed hypothesis as to the difference of change scores between the intervention and control groups, respectively. This should be matched by the use of one-sided tests (Fisher, 1991; Koch and Gillings, 2006), which offer higher power and hence require lower participant numbers. Surprisingly, this simple opportunity for increasing the efficiency of a trial is frequently not taken.

### Alternative Approaches

#### Individual Response

Even for undoubtedly effective interventions (such as exercise training in previously untrained persons) large variability in observed pre–post changes is common (Bouchard et al., 1999; Hecksteden et al., 2015, 2018). From the perspective of verifying (mean) efficacy this variation in observed effects is rather annoying, because it decreases standardized effect sizes and increases the required number of participants. However, when recommending the intervention to individual subjects, the variation in its efficacy between persons is of obvious interest. Importantly, variation in observed pre–post changes does not necessarily reflect interindividual differences in the efficacy of the intervention (“individual response”) but is at least in part due to random variation (e.g., from measurement error and biological day-by-day variability) (Senn, 2004, 2015; Atkinson and Batterham, 2015; Hecksteden et al., 2015). Different methods have been proposed for the quantification of individual response, which in part require specific study designs (Atkinson and Batterham, 2015; Hecksteden et al., 2015, 2018; Senn, 2015). For an RCT, calculating the surplus in variance in the experimental group (random variation plus individual response) as compared to the control group (random variation only) seems to be the most adequate approach (Hecksteden et al., 2018).

Beyond quantifying individual response in terms of a variance or standard deviations, it is appealing to classify individual subjects into responders and non-responders, respectively. However, this classification is beset with theoretical as well as practical difficulties and limitations which are due to two main factors: (i) the unavoidable inaccuracy of the individual response estimate (due to random variation) and (ii) the challenges of identifying a meaningful response threshold (Senn, 2004, 2015; Hecksteden et al., 2018). Therefore, this intuitively tempting approach should be used and interpreted only with great restraint. Moreover, it is important to keep in mind that (despite the simple concept) a considerable number of definitions and operationalizations have been proposed which may result in inconsistent classifications for many individuals (Hecksteden et al., 2018). If the main aim is to have balanced subgroups with marked differences in observed training effects (e.g., for an exploratory search into moderators of training efficacy) a predefined proportion (e.g., 1/3) of individuals with the highest pre–post difference may be labeled as “responders” and a similar proportion of subjects with the lowest pre–post differences as “non-responders” (Timmons et al., 2005). However, such a classification is obviously dependent on the distribution of training effects within the respective trial and may not be interpreted as a general characteristic of the respective subject (Hecksteden et al., 2018). By contrast, if the aim is to characterize individual participants in a meaningful way, the size and uncertainty of an individual’s response (e.g., as confidence interval or effect size) has to be interpreted in relation to prefixed limits of meaningful benefit or harm. In the context of an RCT, uncertainty in the individual pre–post difference may be roughly estimated from the variability of pre–post changes in the control group (Hecksteden et al., 2018).

#### Alternative Approaches to Statistical Inference

While describing the data collected during a specific trial (e.g., participant characteristics, distributions of measured values and calculated indicators such as pre–post differences) is clearly important, the ultimate aim of a scientific study generally is to gain insight into real-world circumstances. Therefore, we need to make inferences from (and therefore beyond) our data. A task which is obviously associated with an unavoidable risk of error.

In recent years, the formerly unrivaled *p*-value based hypothesis-testing approach to inferential statistics has been increasingly criticized (Sterne and Davey Smith, 2001; Wilkinson, 2014; Wasserstein and Lazar, 2016; McShane and Gal, 2017), up to the point of recommending its complete abolishment (Buchheit, 2016). Providing a comprehensive overview of this ongoing controversy including the proposed alternatives (let alone a final judgement) is far beyond the scope of this manuscript. However, a few points should be addressed.

##### What’s the problem with *p*-value based hypothesis testing?

When investigating a specific intervention, we generally want to know whether it is beneficial and can be recommended. Of note, there are two questions involved in this: first, we have to know if the intervention is effective and causes detectable changes in the expected direction at all. If this seems to be the case, we will be interested if the magnitude of the effect is relevant and worth the effort or rather trivially small. In fact, *p*-value based hypothesis testing (at least as it is generally performed in our field) does not provide a direct answer to either of these two questions. Most fundamentally, the *p*-value does not indicate the probability of the intervention being ineffective based on the data analyzed, but the probability of those (or more extreme) data assuming that the intervention is ineffective. While one might intuitively think that the difference between these two conditional probabilities is marginal, there are situations in which the “error of the transposed conditional” becomes most relevant (Wasserstein and Lazar, 2016; Yaddanapudi, 2016).

Moreover, the *p*-value is an amalgamation of effect size (central tendency and variation of the effect) and degrees of freedom (which in most situations are mainly determined by number of subjects). Following the full hypothesis testing logic, the appropriate number of participants for specified type-1 and type-2 error rates has to be calculated in advance and exactly this number has to be studied (Fisher, 1991). If the sample size is larger, even marginal differences will yield “significant” *p*-values. When viewed uncritically, this may lead to the application of interventions which in fact do not cause relevant benefit. There are several other limitations and shortcomings, which have been recently summarized (Wasserstein and Lazar, 2016; Yaddanapudi, 2016).

Several alternative approaches to statistical inference have been devised in response to the shortcomings of *p*-value based hypothesis testing. The most fundamental alternative is Bayesian statistics, a framework in which the concept of probability as a long run relative frequency is replaced by probability as a subjective believe (which has to be refined over and over based on empirical data). There are two important strengths of Bayesian statistics. Most importantly, it allows answering the question we really want to know “How likely is the hypothesis true in consideration of given data?” Secondly, it offers a gradual judgment of the hypothesis being true instead of a dichotomous decision yes or no/significant or non-significant. However, despite the fact that the fundamental shortcomings of *p*-value based hypothesis testing are addressed, truly Bayesian statistics are not yet in mainstream use. One reason may be the subjective notion of probability itself which is counterintuitive for many empirical researchers. More importantly, practical implementation of fully Bayesian analyses is a complex task, which requires learning a new framework of data analysis. There is an increasing number of understandable introductory texts (Kruschke and Liddell, 2018; Quintana and Williams, 2018) but only few scientists seem to master fully Bayesian statistics (Senn, 2011).

As an intermediate solution, approaches based on effect sizes and/or confidence intervals have been proposed. In sport science, magnitude based inferences (MBI) as developed by Hopkins, 2004, Batterham and Hopkins (2006), Hopkins and Batterham (2016) is increasingly popular (Buchheit, 2016). MBI gauges the magnitude of the effect (which is decisive for its practical relevance) and offers a gradual judgment of it being above or below predefined thresholds of practical relevance. However, so far MBI has only been published in sports science journals and personal websites and comprehensive evaluation within the statistical community is still lacking. Moreover, several issues with respect to mathematical as well as conceptual aspects remain controversial (Wilkinson, 2014; Welsh and Knight, 2015; Hopkins and Batterham, 2016; Mengersen et al., 2016; Sainani, 2018; Wilkinson and Winter, 2018). It is beyond the scope of this article to give a final appraisal of MBI. However, publication of a new statistical technique in a recognized statistical journal enabling discussion of its strengths and weaknesses by the expert community should be a matter of course before putting it forth for general use – just as effectiveness of any new exercise training intervention has to be established before transfer to sports practice.

## Conclusion

Exercise training studies should be carefully constructed focusing on the consistency of the whole “package” from an explicit hypothesis or research question over study design and methodology and on the data analysis and interpretation. In doing so, all available information that might affect study power, ideally derived from pilot studies or previously published research should be considered. A clear study question with hypothesis on the primary and secondary endpoints is recommended and should be generated prior to the start of the study. Explorative or uncontrolled trials are only reasonably indicated in pilot or feasibility studies and to state on baseline variability of the primary endpoint, respectively. Validity and reliability of the included methods should be provided for the respective population and age-group. All relevant training characteristics should be thoroughly considered and recorded. Information on training characteristics as detailed as possible should be given within the manuscript.

## Author Contributions

AH, OF, and LD designed the present review. TM contributed to the design. AH, OF, and LD drafted the first manuscript. TM provided notable intellectual input throughout drafting. All authors revised the draft, read and approved the final version of the manuscript.

## Conflict of Interest Statement

The authors declare that the research was conducted in the absence of any commercial or financial relationships that could be construed as a potential conflict of interest.

## Footnotes

1 http://www.who.int/ictrp/network/primary/en/

## References

[B1] Abbiss C. R. Laursen P. B. (2008). Describing and understanding pacing strategies during athletic competition. *Sports Med.* 38 239–252. 10.2165/00007256-200838030-00004 18278984

[B2] Food and Drug Administration (2010). *Guidance for Industry: Adaptive Design Clinical Trials for Drugs and Biologics*. Available at: www.fda.gov/downloads/Drugs/GuidanceComplianceRegulatoryInformation/Guidances/UCM201790.pdf

[B3] Altman D. G. Bland J. M. (2005). Treatment allocation by minimisation. *BMJ* 330:843. 10.1136/bmj.330.7495.843 15817555 PMC556084

[B4] Altman D. G. Moher D. Schulz K. F. (2012). Improving the reporting of randomised trials: the CONSORT Statement and beyond. *Stat. Med.* 31 2985–2997. 10.1002/sim.5402 22903776

[B5] Atkinson G. Batterham A. M. (2015). True and false interindividual differences in the physiological response to an intervention. *Exp. Physiol.* 100 577–588. 10.1113/EP085070 25823596

[B6] Atkinson G. Nevill A. M. (1998). Statistical methods for assessing measurement error (reliability) in variables relevant to sports medicine. *Sports Med.* 26 217–238. 10.2165/00007256-199826040-00002 9820922

[B7] Banister E. W. Calvert T. W. (1980). Planning for future performance: implications for long term training. *Can. J. Appl. Sport Sci.* 5 170–176. 6778623

[B8] Bartlett J. D. O’Connor F. Pitchford N. Torres-Ronda L. Robertson S. J. (2017). Relationships between internal and external training load in team-sport athletes: evidence for an individualized approach. *Int. J. Sports Physiol. Perform.* 12 230–234. 10.1123/ijspp.2015-0791 27194668

[B9] Batterham A. M. Atkinson G. (2005). How big does my sample need to be? A primer on the murky world of sample size estimation. *Phys. Ther. Sport* 6 153–163. 10.1016/j.ptsp.2005.05.004

[B10] Batterham A. M. Hopkins W. G. (2006). Making meaningful inferences about magnitudes. *Int. J. Sports Physiol. Perform.* 1 50–57. 10.1123/ijspp.1.1.50 19114737

[B11] Beedie C. Foad A. Hurst P. (2015). Capitalizing on the placebo component of treatments. *Curr. Sports Med. Rep.* 14 284–287. 10.1249/JSR.0000000000000172 26166052

[B12] Beedie C. Foad A. J. (2009). The placebo effect in sports performance: a brief review. *Sports Med.* 39 313–329. 10.2165/00007256-200939040-00004 19317519

[B13] Berdi M. Koteles F. Szabo A. Bardos G. (2011). Placebo effects in sport and exercise: a meta-analysis. *Eur. J. Ment. Health* 6 196–212. 10.5708/EJMH.6.2011.2.5

[B14] Bouchard C. An P. Rice T. Skinner J. S. Wilmore J. H. Gagnon J. (1999). Familial aggregation of VO(2max) response to exercise training: results from the HERITAGE family study. *J. Appl. Physiol.* 87 1003–1008. 10.1152/jappl.1999.87.3.1003 10484570

[B15] Broatch J. R. Petersen A. Bishop D. J. (2014). Postexercise cold water immersion benefits are not greater than the placebo effect. *Med. Sci. Sports Exerc.* 46 2139–2147. 10.1249/MSS.0000000000000348 24674975

[B16] Buchheit M. (2016). The numbers will love you back in return-I promise. *Int. J. Sports Physiol. Perform.* 11 551–554. 10.1123/IJSPP.2016-0214 27164726

[B17] Burgomaster K. A. Howarth K. R. Phillips S. M. Rakobowchuk M. Macdonald M. J. McGee S. L. (2008). Similar metabolic adaptations during exercise after low volume sprint interval and traditional endurance training in humans. *J. Physiol.* 586 151–160. 10.1113/jphysiol.2007.142109 17991697 PMC2375551

[B18] Campbell M. K. Piaggio G. Elbourne D. R. Altman D. G. Group C. (2012). Consort 2010 statement: extension to cluster randomised trials. *BMJ* 345:e5661. 10.1136/bmj.e5661 22951546

[B19] Chodzko-Zajko W. J. Proctor D. N. Fiatarone Singh M. A. Minson C. T. Nigg C. R. Salem G. J. (2009). American college of sports medicine position stand. Exercise and physical activity for older adults. *Med. Sci. Sports Exerc.* 41 1510–1530. 10.1249/MSS.0b013e3181a0c95c 19516148

[B20] Coyle E. F. (1995). Integration of the physiological factors determining endurance performance ability. *Exerc. Sport Sci. Rev.* 23 25–63. 10.1249/00003677-199500230-00004 7556353

[B21] Currell K. Jeukendrup A. E. (2008). Validity, reliability and sensitivity of measures of sporting performance. *Sports Med.* 38 297–316. 10.2165/00007256-200838040-00003 18348590

[B22] Dixon P. M. Saint-Maurice P. F. Kim Y. Hibbing P. Bai Y. Welk G. J. (2018). A primer on the use of equivalence testing for evaluating measurement agreement. *Med. Sci. Sports Exerc.* 50 837–845. 10.1249/MSS.0000000000001481 29135817 PMC5856600

[B23] Donath L. Faude O. Roth R. Zahner L. (2014). Effects of stair-climbing on balance, gait, strength, resting heart rate, and submaximal endurance in healthy seniors. *Scand. J. Med. Sci. Sports* 24 e93–e101. 10.1111/sms.12113 24033611

[B24] Donath L. Rossler R. Faude O. (2016a). Effects of virtual reality training (Exergaming) compared to alternative exercise training and passive control on standing balance and functional mobility in healthy community-dwelling seniors: a meta-analytical review. *Sports Med.* 46 1293–1309. 10.1007/s40279-016-0485-1 26886474

[B25] Donath L. Roth R. Zahner L. Faude O. (2017). Slackline training (balancing over narrow nylon ribbons) and balance performance: a meta-analytical review. *Sports Med.* 47 1075–1086. 10.1007/s40279-016-0631-9 27704483

[B26] Donath L. van Dieen J. Faude O. (2016b). Exercise-based fall prevention in the elderly: What about agility? *Sports Med.* 46 143–149. 10.1007/s40279-015-0389-5 26395115

[B27] Eldridge S. M. Chan C. L. Campbell M. J. Bond C. M. Hopewell S. Thabane L. (2016). CONSORT 2010 statement: extension to randomised pilot and feasibility trials. *BMJ* 355:i5239. 10.1136/bmj.i5239 27777223 PMC5076380

[B28] Faude O. Kindermann W. Meyer T. (2009). Lactate threshold concepts: How valid are they? *Sports Med.* 39 469–490. 10.2165/00007256-200939060-00003 19453206

[B29] Faude O. Rössler R. Petushek E. J. Roth R. Zahner L. Donath L. (2017). Neuromuscular adaptations to multimodal injury prevention programs in youth sports: a systematic review with meta-analysis of randomized controlled trials. *Front. Physiol.* 8:791. 10.3389/fphys.2017.00791 29075200 PMC5643472

[B30] Faude O. Schnittker R. Schulte-Zurhausen R. Muller F. Meyer T. (2013). High intensity interval training vs. high-volume running training during pre-season conditioning in high-level youth football: a cross-over trial. *J. Sports Sci.* 31 1441–1450. 10.1080/02640414.2013.792953 23725006

[B31] Faude O. Steffen A. Kellmann M. Meyer T. (2014). The effect of short-term interval training during the competitive season on physical fitness and signs of fatigue: a crossover trial in high-level youth football players. *Int. J. Sports Physiol. Perform.* 9 936–944. 10.1123/ijspp.2013-0429 24622685

[B32] Finch C. F. Donaldson A. (2010). A sports setting matrix for understanding the implementation context for community sport. *Br. J. Sports Med.* 44 973–978. 10.1136/bjsm.2008.056069 19201766

[B33] Fisher L. (1991). The use of one-sided tests in drug trials: an FDA advisory committee member’s perspective. *J. Biopharm. Stat.* 1 151–156. 10.1080/10543409108835012 1844686

[B34] Foster C. Richards J. Thorogood M. Hillsdon M. (2013). Remote and web 2.0 interventions for promoting physical activity. *Cochrane Database Syst. Rev.* 9:CD010395. 10.1002/14651858.CD010395.pub2 PMC9674455 24085594

[B35] Foster C. Rodriguez-Marroyo J. A. de Koning J. J. (2017). Monitoring training loads: the past, the present, and the future. *Int. J. Sports Physiol. Perform.* 12 S22–S28. 10.1123/ijspp.2016-0388 28253038

[B36] Fyfe J. J. Bishop D. J. Stepto N. K. (2014). Interference between concurrent resistance and endurance exercise: molecular bases and the role of individual training variables. *Sports Med.* 44 743–762. 10.1007/s40279-014-0162-1 24728927

[B37] Garber C. E. Blissmer B. Deschenes M. R. Franklin B. A. Lamonte M. J. Lee I. M. (2011). American college of sports medicine position stand. Quantity and quality of exercise for developing and maintaining cardiorespiratory, musculoskeletal, and neuromotor fitness in apparently healthy adults: guidance for prescribing exercise. *Med. Sci. Sports Exerc.* 43 1334–1359. 10.1249/MSS.0b013e318213fefb 21694556

[B38] Gibala M. J. Little J. P. van Essen M. Wilkin G. P. Burgomaster K. A. Safdar A. (2006). Short-term sprint interval versus traditional endurance training: similar initial adaptations in human skeletal muscle and exercise performance. *J. Physiol.* 575 901–911. 10.1113/jphysiol.2006.112094 16825308 PMC1995688

[B39] Giboin L. S. Gruber M. Kramer A. (2015). Task-specificity of balance training. *Hum. Mov. Sci.* 44 22–31. 10.1016/j.humov.2015.08.012 26298214

[B40] Gilchrist J. Mandelbaum B. R. Melancon H. Ryan G. W. Silvers H. J. Griffin L. Y. (2008). A randomized controlled trial to prevent noncontact anterior cruciate ligament injury in female collegiate soccer players. *Am. J. Sports Med.* 36 1476–1483. 10.1177/0363546508318188 18658019

[B41] Granata C. Oliveira R. S. Little J. P. Renner K. Bishop D. J. (2016). Mitochondrial adaptations to high-volume exercise training are rapidly reversed after a reduction in training volume in human skeletal muscle. *FASEB J.* 30 3413–3423. 10.1096/fj.201500100R 27402675

[B42] Haley S. M. Fragala-Pinkham M. A. (2006). Interpreting change scores of tests and measures used in physical therapy. *Phys. Ther.* 86 735–743. 16649896

[B43] Harriss D. J. Atkinson G. (2015). Ethical standards in sport and exercise science research: 2016 update. *Int. J. Sports Med.* 36 1121–1124. 10.1055/s-0035-1565186 26671845

[B44] Hawley J. A. (2009). Molecular responses to strength and endurance training: are they incompatible? *Appl. Physiol. Nutr. Metab.* 34 355–361. 10.1139/H09-023 19448698

[B45] Haynes R. B. Ackloo E. Sahota N. McDonald H. P. Yao X. (2008). Interventions for enhancing medication adherence. *Cochrane Database Syst. Rev.* CD000011. 10.1002/14651858.CD000011.pub3 18425859

[B46] Hecksteden A. Kraushaar J. Scharhag-Rosenberger F. Theisen D. Senn S. Meyer T. (2015). Individual response to exercise training - a statistical perspective. *J. Appl. Physiol.* 118 1450–1459. 10.1152/japplphysiol.00714.2014 25663672

[B47] Hecksteden A. Pitsch W. Rosenberger F. Meyer T. (2018). Repeated testing for the assessment of individual response to exercise training. *J. Appl. Physiol.* 124 1567–1579. 10.1152/japplphysiol.00896.2017 29357481

[B48] Hecksteden A. Wegmann M. Steffen A. Kraushaar J. Morsch A. Ruppenthal S. (2013). Irisin and exercise training in humans - Results from a randomized controlled training trial. *BMC Med* 11:235. 10.1186/1741-7015-11-235 24191966 PMC4228275

[B49] Helgerud J. Hoydal K. Wang E. Karlsen T. Berg P. Bjerkaas M. (2007). Aerobic high-intensity intervals improve VO2max more than moderate training. *Med. Sci. Sports Exerc.* 39 665–671. 10.1249/mss.0b013e3180304570 17414804

[B50] Hertogh E. M. Schuit A. J. Peeters P. H. Monninkhof E. M. (2010). Noncompliance in lifestyle intervention studies: the instrumental variable method provides insight into the bias. *J. Clin. Epidemiol.* 63 900–906. 10.1016/j.jclinepi.2009.10.007 20189770

[B51] Hopewell S. Dutton S. Yu L. M. Chan A. W. Altman D. G. (2010). The quality of reports of randomised trials in 2000 and 2006: comparative study of articles indexed in PubMed. *BMJ* 340:c723. 10.1136/bmj.c723 20332510 PMC2844941

[B52] Hopkins W. G. (2000). Measures of reliability in sports medicine and science. *Sports Med.* 30 1–15. 10.2165/00007256-200030010-00001 10907753

[B53] Hopkins W. G. (2004). How to interpret changes in an athletic performance test. *Sportscience* 8 1–7.

[B54] Hopkins W. G. (2006). Estimating sample size for magnitude-based inferences. *Sportscience* 10 63–70.

[B55] Hopkins W. G. (2015). Individual responses made easy. *J. Appl. Physiol.* 118 1444–1446. 10.1152/japplphysiol.00098.2015 25678695

[B56] Hopkins W. G. Batterham A. M. (2016). Error rates, decisive outcomes and publication bias with several inferential methods. *Sports Med.* 46 1563–1573. 10.1007/s40279-016-0517-x 26971328

[B57] Koch G. G. Gillings D. B. (2006). *Encyclopedia of Statistical Sciences.* Hoboken, NJ: John Wiley & Sons, Inc.

[B58] Kruschke J. K. Liddell T. M. (2018). Bayesian data analysis for newcomers. *Psychon. Bull. Rev.* 25 155–177. 10.3758/s13423-017-1272-1 28405907

[B59] Kummel J. Kramer A. Giboin L. S. Gruber M. (2016). Specificity of balance training in healthy individuals: a systematic review and meta-analysis. *Sports Med.* 46 1261–1271. 10.1007/s40279-016-0515-z 26993132

[B60] Lakens D. (2017). Equivalence tests: a practical primer for t tests, correlations, and meta-analyses. *Soc. Psychol. Personal. Sci.* 8 355–362. 10.1177/1948550617697177 28736600 PMC5502906

[B61] Lakens D. McLatchie N. Isager P. M. Scheel A. M. Dienes Z. (2018). Improving inferences about null effects with bayes factors and equivalence tests. *J. Gerontol. B Psychol. Sci. Soc. Sci.* 10.1093/geronb/gby065 [Epub ahead of print]. 29878211

[B62] Lambert M. I. Borresen J. (2010). Measuring training load in sports. *Int. J. Sports Physiol. Perform.* 5 406–411. 10.1123/ijspp.5.3.406 20861529

[B63] Lesinski M. Hortobagyi T. Muehlbauer T. Gollhofer A. Granacher U. (2015). Effects of balance training on balance performance in healthy older adults: a systematic review and meta-analysis. *Sports Med.* 45 1721–1738. 10.1007/s40279-015-0375-y 26325622 PMC4656699

[B64] Lindquist R. Wyman J. F. Talley K. M. Findorff M. J. Gross C. R. (2007). Design of control-group conditions in clinical trials of behavioral interventions. *J. Nurs. Scholarsh.* 39 214–221. 10.1111/j.1547-5069.2007.00171.x 17760793

[B65] Mandelbaum B. R. Silvers H. J. Watanabe D. S. Knarr J. F. Thomas S. D. Griffin L. Y. (2005). Effectiveness of a neuromuscular and proprioceptive training program in preventing anterior cruciate ligament injuries in female athletes: 2-year follow-up. *Am. J. Sports Med.* 33 1003–1010. 10.1177/0363546504272261 15888716

[B66] McKay C. D. Verhagen E. (2016). ‘Compliance’ versus ‘adherence’ in sport injury prevention: why definition matters. *Br. J. Sports Med.* 50 382–383. 10.1136/bjsports-2015-095192 26682865

[B67] McPhate L. Simek E. M. Haines T. P. (2013). Program-related factors are associated with adherence to group exercise interventions for the prevention of falls: a systematic review. *J. Physiother.* 59 81–92. 10.1016/S1836-9553(13)70160-7 23663793

[B68] McShane B. B. Gal D. (2017). Statistical significance and the dichotomization of evidence. *J. Am. Stat. Assoc.* 112 885–895. 10.1080/01621459.2017.1289846 PMC5769160 29348701

[B69] Mengersen K. L. Drovandi C. C. Robert C. P. Pyne D. B. Gore C. J. (2016). Bayesian estimation of small effects in exercise and sports science. *PLoS One* 11:e0147311. 10.1371/journal.pone.0147311 27073897 PMC4830602

[B70] Meyer T. Auracher M. Heeg K. Urhausen A. Kindermann W. (2006). Does cumulating endurance training at the weekends impair training effectiveness? *Eur. J. Cardiovasc. Prev. Rehabil.* 13 578–584. 10.1097/01.hjr.0000198921.34814.4d 16874148

[B71] Meyer T. Scharhag J. Kindermann W. (2005). Peak oxygen uptake. Myth and truth about an internationally accepted reference value. *Z. Kardiol.* 94 255–264. 10.1007/s00392-005-0207-4 15803262

[B72] Moher D. Hopewell S. Schulz K. F. Montori V. Gotzsche P. C. Devereaux P. J. (2010). CONSORT 2010 explanation and elaboration: updated guidelines for reporting parallel group randomised trials. *BMJ* 340:c869. 10.1136/bmj.c869 20332511 PMC2844943

[B73] O’Brien J. Finch C. F. (2014). The implementation of musculoskeletal injury-prevention exercise programmes in team ball sports: a systematic review employing the RE-AIM framework. *Sports Med.* 44 1305–1318. 10.1007/s40279-014-0208-4 24986117

[B74] O’Donovan G. Lee I. M. Hamer M. Stamatakis E. (2017). Association of “Weekend Warrior” and other leisure time physical activity patterns with risks for all-cause, cardiovascular disease, and cancer mortality. *JAMA Intern. Med.* 177 335–342. 10.1001/jamainternmed.2016.8014 28097313

[B75] Piaggio G. Elbourne D. R. Pocock S. J. Evans S. J. Altman D. G. Group C. (2012). Reporting of noninferiority and equivalence randomized trials: extension of the CONSORT 2010 statement. *JAMA* 308 2594–2604. 10.1001/jama.2012.87802 23268518

[B76] Pocock S. J. Simon R. (1975). Sequential treatment assignment with balancing for prognostic factors in the controlled clinical trial. *Biometrics* 31 103–115. 10.2307/2529712 1100130

[B77] Quintana D. Williams D. (2018). Bayesian alternatives for common null-hypothesis significance tests in psychiatry: a non-technical guide using JASP. *BMC Psychiatry* 18:178. 10.1186/s12888-018-1761-4 29879931 PMC5991426

[B78] Rodrigues A. L. Ball J. Ski C. Stewart S. Carrington M. J. (2016). A systematic review and meta-analysis of primary prevention programmes to improve cardio-metabolic risk in non-urban communities. *Prev. Med.* 87 22–34. 10.1016/j.ypmed.2016.02.011 26876624

[B79] Rosenberger W. F. Sverdlov O. Hu F. (2012). Adaptive randomization for clinical trials. *J. Biopharm. Stat.* 22 719–736. 10.1080/10543406.2012.676535 22651111

[B80] Sainani K. L. (2018). The problem with “Magnitude-based inference”. *Med. Sci. Sports Exerc.* 10.1249/MSS.0000000000001645 [Epub ahead of print]. 29683920

[B81] Scharhag-Rosenberger F. Walitzek S. Kindermann W. Meyer T. (2012). Differences in adaptations to 1 year of aerobic endurance training: individual patterns of nonresponse. *Scand. J. Med. Sci. Sports* 22 113–118. 10.1111/j.1600-0838.2010.01139.x 20561283

[B82] Schulz K. F. Altman D. G. Moher D. Group C. (2010). CONSORT 2010 statement: updated guidelines for reporting parallel group randomised trials. *BMJ* 340:c332. 10.1136/bmj.c332 20332509 PMC2844940

[B83] Schulz K. F. Grimes D. A. (2002). Blinding in randomised trials: hiding who got what. *Lancet* 359 696–700. 10.1016/S0140-6736(02)07816-9 11879884

[B84] Scott N. W. McPherson G. C. Ramsay C. R. Campbell M. K. (2002). The method of minimization for allocation to clinical trials. a review. *Control. Clin. Trials* 23 662–674. 10.1016/S0197-2456(02)00242-8 12505244

[B85] Sedgwick P. Hoope C. (2014). Placebo controlled trials. *BMJ* 348:g1635. 10.1136/bmj.g1635 24563452

[B86] Seiler S. (2010). What is best practice for training intensity and duration distribution in endurance athletes? *Int. J. Sports Physiol. Perform.* 5 276–291. 10.1123/ijspp.5.3.276 20861519

[B87] Senn S. (1995). Base logic: tests of baseline balance in randomized clinical trials. *Clin. Res. Regul. Affairs* 12 171–182. 10.3109/10601339509019426

[B88] Senn S. (2004). Individual response to treatment: is it a valid assumption? *BMJ* 329 966–968. 10.1136/bmj.329.7472.966 15499115 PMC524113

[B89] Senn S. (2009). Three things that every medical writer should know about statistics. *Write Stuff* 18 159–162.

[B90] Senn S. (2011). You may believe you are a bayesian but you are probably wrong. *RMM* 2 48–66.

[B91] Senn S. (2013). Seven myths of randomisation in clinical trials. *Stat. Med.* 32 1439–1450. 10.1002/sim.5713 23255195

[B92] Senn S. (2015). Mastering variation: variance components and personalised medicine. *Stat. Med.* 35 966–977. 10.1002/sim.6739 26415869 PMC5054923

[B93] Sherrington C. Michaleff Z. A. Fairhall N. Paul S. S. Tiedemann A. Whitney J. (2017). Exercise to prevent falls in older adults: an updated systematic review and meta-analysis. *Br. J. Sports Med.* 51 1750–1758. 10.1136/bjsports-2016-096547 27707740

[B94] Sherrington C. Tiedemann A. Fairhall N. Close J. C. Lord S. R. (2011). Exercise to prevent falls in older adults: an updated meta-analysis and best practice recommendations. *N. S. W. Public Health Bull.* 22 78–83. 10.1071/NB10056 21632004

[B95] Shiroma E. J. Lee I. M. (2018). Can we proceed with physical activity recommendations if (almost) no clinical trial data exist on mortality? *Br. J. Sports Med.* 52 888–889. 10.1136/bjsports-2018-099185 29545235 PMC6100792

[B96] Simek E. M. McPhate L. Haines T. P. (2012). Adherence to and efficacy of home exercise programs to prevent falls: a systematic review and meta-analysis of the impact of exercise program characteristics. *Prev. Med.* 55 262–275. 10.1016/j.ypmed.2012.07.007 22813920

[B97] Sjosten N. M. Salonoja M. Piirtola M. Vahlberg T. J. Isoaho R. Hyttinen H. K. (2007). A multifactorial fall prevention programme in the community-dwelling aged: predictors of adherence. *Eur. J. Public Health* 17 464–470. 10.1093/eurpub/ckl272 17208952

[B98] Slade S. C. Dionne C. E. Underwood M. Buchbinder R. (2016). Consensus on exercise reporting template (CERT): explanation and elaboration statement. *Br. J. Sports Med.* 96 1514–1524. 10.1136/bjsports-2016-096651 27707738

[B99] Sterne J. A. Davey Smith G. (2001). Sifting the evidence-what’s wrong with significance tests? *BMJ* 322 226–231. 11159626 10.1136/bmj.322.7280.226 PMC1119478

[B100] Stöggl T. Sperlich B. (2015). The training intensity distribution among well-trained and elite endurance athletes. *Front. Physiol.* 6:295 10.3389/fphys.2015.00295 PMC4621419 26578968

[B101] Thiese M. S. (2014). Observational and interventional study design types; an overview. *Biochem. Med.* 24 199–210. 10.11613/BM.2014.022 24969913 PMC4083571

[B102] Timmons J. A. Jansson E. Fischer H. Gustafsson T. Greenhaff P. L. Ridden J. (2005). Modulation of extracellular matrix genes reflects the magnitude of physiological adaptation to aerobic exercise training in humans. *BMC Biol.* 3:19. 10.1186/1741-7007-3-19 16138928 PMC1224855

[B103] Toraman N. F. (2005). Short term and long term detraining: is there any difference between young-old and old people? *Br. J. Sports Med.* 39 561–564. 10.1136/bjsm.2004.015420 16046344 PMC1725295

[B104] Toraman N. F. Ayceman N. (2005). Effects of six weeks of detraining on retention of functional fitness of old people after nine weeks of multicomponent training. *Br. J. Sports Med.* 39 565–568. 10.1136/bjsm.2004.015586 16046345 PMC1725289

[B105] Treasure T. MacRae K. D. (1998). Minimisation: the platinum standard for trials?. Randomisation doesn’t guarantee similarity of groups; minimisation does. *BMJ* 317 362–363. 10.1136/bmj.317.7155.362 9694748 PMC1113668

[B106] van Breukelen G. J. Candel M. J. (2012). Calculating sample sizes for cluster randomized trials: we can keep it simple and efficient! *J. Clin. Epidemiol.* 65 1212–1218. 10.1016/j.jclinepi.2012.06.002 23017638

[B107] Verhagen E. Bolling C. Finch C. F. (2015). Caution this drug may cause serious harm! Why we must report adverse effects of physical activity promotion. *Br. J. Sports Med.* 49 1–2. 10.1136/bjsports-2014-093604 25082617

[B108] Vohra S. Shamseer L. Sampson M. Bukutu C. Schmid C. H. Tate R. (2015). CONSORT extension for reporting N-of-1 trials (CENT) 2015 Statement. *BMJ* 350:h1738. 10.1136/bmj.h1738 25976398

[B109] Wasserstein R. Lazar N. (2016). The ASA’s statement on p-values: context, process, and purpose. *Am. Stat.* 70 129–133. 10.1080/00031305.2016.1154108

[B110] Welsh A. H. Knight E. J. (2015). “Magnitude-based inference”: a statistical review. *Med. Sci. Sports Exerc.* 47 874–884. 10.1249/MSS.0000000000000451 25051387 PMC5642352

[B111] White A. Ernst E. (2001). The case for uncontrolled clinical trials: a starting point for the evidence base for CAM. *Complement. Ther. Med.* 9 111–116. 10.1054/ctim.2001.0441 11444891

[B112] Wilkerson G. B. Denegar C. R. (2014). Cohort study design: an underutilized approach for advancement of evidence-based and patient-centered practice in athletic training. *J. Athl. Train.* 49 561–567. 10.4085/1062-6050-49.3.43 24933432 PMC4151846

[B113] Wilkinson M. (2014). Distinguishing between statistical significance and practical/clinical meaningfulness using statistical inference. *Sports Med.* 44 295–301. 10.1007/s40279-013-0125-y 24248505

[B114] Wilkinson M. Winter E. M. (2018). Estimation versus falsification approaches in sport and exercise science. *J. Sports Sci.* 10.1080/02640414.2018.1479116 [Epub ahead of print]. 29786469

[B115] World Health Organisation (2003). *Adherance to Long-Term Therapies: Evidence for Action*. Available at: http://www.who.int/chp/knowledge/publications/adherence_report/en/

[B116] World Medical Association (2013). World medical association declaration of Helsinki: ethical principles for medical research involving human subjects. *JAMA* 310 2191–2194. 10.4103/0970-9185.194772 24141714

[B117] Yaddanapudi L. N. (2016). The American statistical association statement on P-values explained. *J. Anaesthesiol. Clin. Pharmacol.* 32 421–423. 28096569 10.4103/0970-9185.194772 PMC5187603

